# Efficacy and prognostic impact of preoperative risk factors for salvage liver transplantation and repeat hepatectomy in patients with early-stage recurrent hepatocellular carcinoma: a propensity score-matched analysis

**DOI:** 10.3389/fonc.2025.1547054

**Published:** 2025-02-24

**Authors:** Linfeng Yang, Yang Huang, Dawei Deng, Junning Liu, Liangliang Xu, Pengsheng Yi

**Affiliations:** ^1^ Department of Hepato-Biliary-Pancrease II, Affiliated Hospital of North Sichuan Medical College, Nanchong, China; ^2^ Department of Liver Surgery, Liver Transplantation Center, West China Hospital of Sichuan University, Chengdu, China; ^3^ Department of Cardiovascular Surgery, Beijing Anzhen Nanchong Hospital of Capital Medical University & Nanchong Central Hospital, The Second Clinical Medical College of North Sichuan Medical College, Nanchong, China

**Keywords:** recurrent hepatocellular, salvage liver transplantation, repeat hepatectomy, survival outcome, prognostic index

## Abstract

**Background:**

The optimal treatment strategy for recurrent hepatocellular carcinoma (rHCC) remains unclear. This study is based on cases of rHCC after liver resection, aiming to evaluate the influence of preoperative risk factors on the long-term prognosis of patients with rHCC by comparing patients who underwent salvage liver transplantation (SLT) with those who underwent repeat hepatectomy (RH).

**Methods:**

We retrospectively analyzed 401 consecutive patients with rHCC who underwent SLT or RH between March 2015 and December 2022. Next, we performed propensity score matching, subgroup analyses, and both univariate and multivariate analyses. In addition, Kaplan–Meier analysis was used to estimate the overall survival (OS) and recurrence-free survival (RFS) after recurrence.

**Results:**

The 1-, 3-, and 5-year OS and RFS rates in the SLT group were significantly higher than those in the RH group (p=0.0131 and p=0.0010, respectively), and similar results were observed after propensity score matching. In the presence of zero or one risk factors, the OS and RFS in the SLT group were significantly better than those in the RH group (p=0.0386 and p=0.0117, respectively). However, in the presence of two to four risk factors, no significant differences in OS or RFS were detected between the two groups (p=0.1119 and p=0.1035, respectively).

**Conclusion:**

Our analysis identified a number of risk factors that were strongly correlated with a long term prognosis for patients with rHCC who underwent SLT and RH: multiple tumors, a maximum tumor diameter ≥5 cm, microvascular invasion, and a recurrence time ≤2 years. Our findings provide important reference guidelines for organ allocation and clinical decision-making.

## 1 Introduction

Hepatocellular carcinoma (HCC) is one of the most common malignancies, accounting for 90% of all primary liver cancers and representing the predominant pathological type (1, 2). Globally, HCC is the third leading cause of all cancer-related deaths and primarily affects patients with chronic liver disease; furthermore, the incidence of HCC is increasing on an annual basis (2, 3). Liver transplantation (LT) and liver resection (LR) are the primary curative treatment options for HCC. Theoretically, LT represents the optimal treatment for HCC as it removes both the tumor and underlying liver disease, yielding a 5-year postoperative survival rate of 75% and a recurrence rate of 20% (4, 5). However, owing to donor shortages and the risk of tumor progression, LR is more commonly performed and has become the mainstay treatment for HCC, particularly for patients with early stage disease who meet specific criteria (6, 7). LR can significantly extend survival in patients with early stage HCC, with a 5-year survival rate of 50% but a high recurrence rate of 70% (8–10). The application of LR is also increasing in patients with advanced HCC, localized lesions, and preserved liver function (11). Nevertheless, owing to the chronic liver disease and cirrhotic background of patients with HCC, approximately 80% of cases experience intrahepatic recurrence following surgery, with typically smaller recurrent tumors than primary tumors (7, 12, 13).

The treatment of recurrent hepatocellular carcinoma (rHCC) is crucial because of the high recurrence rate following LR. The current primary curative treatments for rHCC include salvage liver transplantation (SLT), repeat hepatectomy (RH), and radiofrequency ablation (RFA). SLT has been proposed as a strategy to conserve liver resources and mitigate the risks of transplantation. SLT refers to the treatment strategy of LT when liver cancer relapses or liver failure occurs after hepatectomy, which may alleviate the shortage of donors and expand the treatment opportunities for patients on the waiting list (14). However, significant variations in the long-term survival outcomes of patients following SLT have been observed among patients meeting the Milan criteria across regions such as Asia and Europe; furthermore, these variations have been recorded in subgroups based on the timing of tumor recurrence, the levels of alpha-fetoprotein, and the status of liver injury (15–17). While RH is frequently used to treat rHCC, this technique is limited by a range of factors, including a small residual liver volume, an inadequate reserve of liver functionality, multiple recurrences, and abdominal adhesions (18, 19). RFA, as a localized form of treatment, offers a level of efficacy similar to that of RH, while preserving liver function (20).

Currently, there are no standardized guidelines for the application of SLT, and survival outcomes vary significantly across different research centers, resulting in the usage of donor livers. In the present study, we aimed to identify the prognostic risk factors that influence survival in patients undergoing SLT based on the initial surgical approach and the specific clinicopathological features of rHCC recurrence, evaluate the suitability of SLT as a clinical procedure, and provide clinical guidance for optimizing liver allocation policies.

## 2 Methods

### 2.1 Study population

This retrospective study included patients with rHCC who underwent SLT or RH at the Department of Hepatobiliary Surgery and Transplant Center of West China Hospital, Sichuan University, between March 2015 and December 2022.All participants were over 18 years old and had a pathological diagnosis of HCC, meeting the University of California, San Francisco (UCSF) criteria: a single tumor with a maximum diameter of ≤6.5 cm; ≤3 tumors, each with a diameter of ≤4.5 cm, and a total diameter ≤8 cm; no major vascular invasion or extrahepatic metastasis. Before RH, the patient did not receive anti-tumor therapy; during the waiting period for SLT, some patients received interventional embolization, targeted therapy and immunotherapy. After RH and SLT, some patients received interventional embolization, targeted therapy and immunotherapy. Exclusion criteria included significant comorbidities at the time of recurrence (such as heart, lung, or liver/kidney failure) or loss of surgical opportunity due to tumor progression. All SLT were from cadaveric donors. This study was conducted in accordance with the Declaration of Helsinki (1975) and was approved by the Ethics Committee of West China Hospital. Informed consent was obtained from all patients.

### 2.2 Preoperative assessment

Demographic characteristics included age, sex, body mass index (BMI), and cirrhosis etiology. Tumor characteristics, including size, number, major vascular invasion, and distant metastasis, were assessed preoperatively by computed tomography (CT) and magnetic resonance imaging (MRI), whereas hepatic blood flow was evaluated by ultrasonography. Tumor histological type, grade, and invasion depth were assessed by pathological examination. Liver function was evaluated by the model for end-stage liver disease (MELD) score, Child-Pugh score, serological tests, and the indocyanine green (ICG) test. Patients with severe diseases of the heart, lungs, brain, or kidneys, as well as those with extrahepatic tumor metastasis, were excluded. Patients classified as Child-Pugh grade A, or those restored to grade A following treatment, were eligible for RH if the 15-minute ICG test results were normal and the predicted residual liver volume exceeded 30%. SLT was selected for patients who met the UCSF criteria and had been evaluated by the MELD score (MELD >18: high risk; 15–18: medium risk; ≤14: low risk).

### 2.3 Diagnostic criteria and definitions

The clinical diagnosis of HCC, both at the initial and recurrent stages, was considered to be reliable if a given patient met the American Association for the Study of Liver Diseases criteria, was confirmed by histopathology, and the diagnosis aligned with CT and MRI findings. Suspected lesions were biopsied under ultrasonographic guidance. Overall survival (OS) is defined as the time from the RH or SLT to the patient’s death or the end of the follow-up period. Recurrence-free survival (RFS) was defined as the time from treatment for rHCC to the date of recurrence.

### 2.4 Patient follow-up

Owing to the high recurrence rate of HCC following LR, patients were followed-up every 3 months during the first year after discharge, bi-weekly from 3–6 months, and monthly thereafter. Follow-up assessments included blood tests, liver and kidney function evaluations, tumor markers (alpha-fetoprotein and abnormal prothrombin levels), and ultrasound examinations. CT and MRI scans were conducted every 3 months during the first year and subsequently every 3–6 months. Patients were readmitted for tumor recurrence, liver dysfunction, or other emergencies, as required. The study endpoint was defined as loss to follow-up, death, or by the final date of the study (December 31, 2022).

### 2.5 Statistical analysis

Continuous variables are expressed as median (range), while categorical variables are presented as percentages. The significance of differences between continuous variables was determined using Student’s t-test or the Mann-Whitney U test, while categorical variables were assessed using the chi-square test or Fisher’s exact test. The 5-year OS was the primary endpoint, and the 5-year RFS was the secondary endpoint. Kaplan-Meier curves were used to generate survival estimates, and the log-rank test was applied for comparisons. Univariate Cox regression analysis identified relevant factors, and hazard ratios (HRs) with 95% confidence intervals (CIs) were reported. Multivariate analysis included variables that were significant in univariate analysis (p < 0.05). Statistical significance was set at p < 0.05. Data analysis was performed using SPSS (version 26.0) and GraphPad Prism (version 9.5).To minimize confounding biases between the SLT and RH groups (including age, gender, hepatitis background, liver function index, platelet count, international normalized ratio, Child-Pugh classification, etc.), propensity score matching (PSM, caliper value 0.02) was performed using a 1:3 matching ratio with R software (version 4.41). The standardized mean differences (SMD) before and afterPSM were calculated to measure balance between groups.

## 3 Results

### 3.1 Baseline data at recurrence

**Figure 1:**
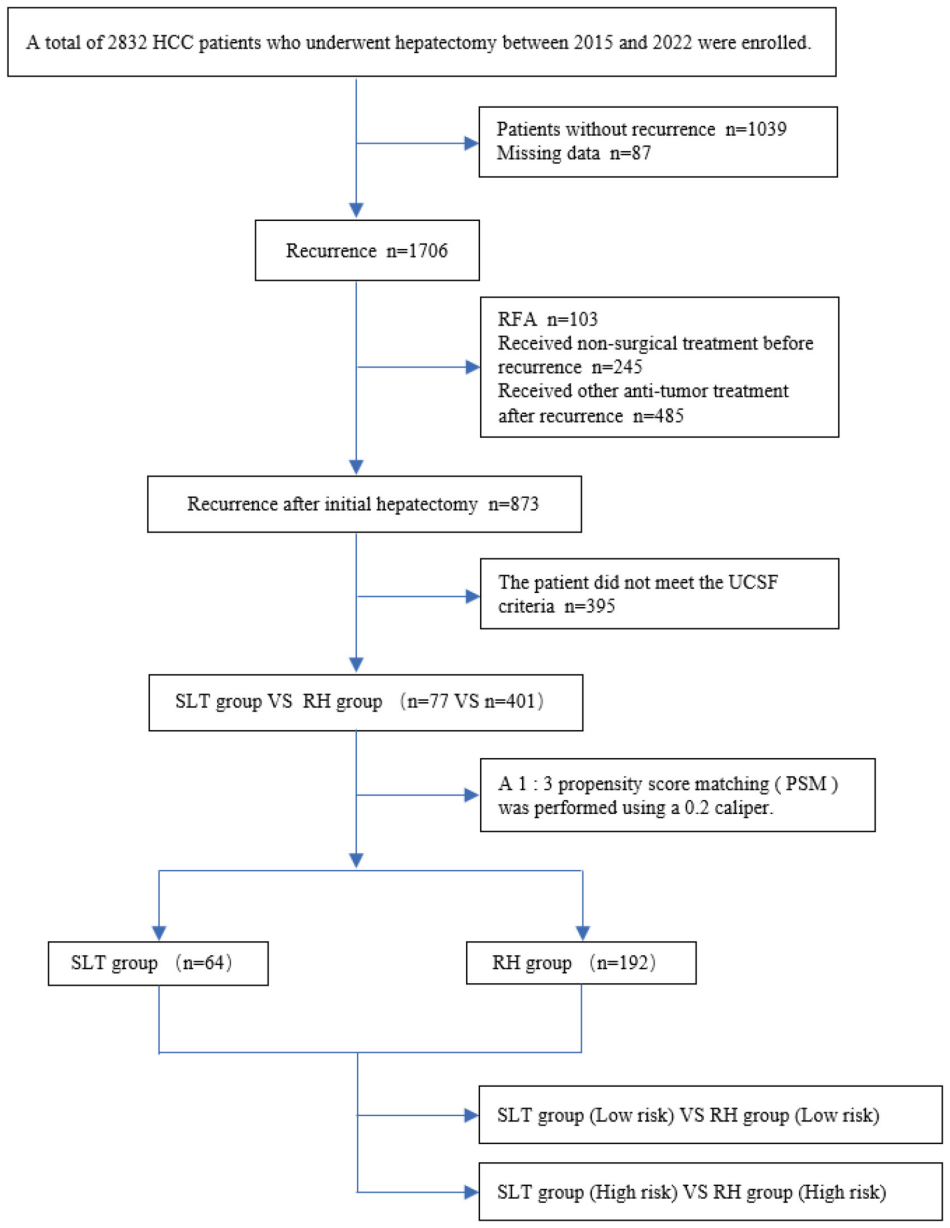
Flow chart depicting the study. HCC, hepatocellular carcinoma; RFA, radiofrequency ablation; SLT, salvage liver transplantation; RH, repeated hepatectomy.

**Figure 1** presents a flowchart describing this study. Between 2015 and 2022, a total of 2,832 patients with HCC underwent liver resection at the West China Hospital. During follow-up, 1,039 patients (36.7%) did not experience tumor recurrence, 36 (3.1%) were lost to follow-up, and 1,706 (60.2%) experienced recurrence. Of the patients experiencing recurrence, 103 underwent RFA, 245 received non-surgical treatment prior to recurrence, and 485 received other antitumor therapies post-recurrence. We excluded 395 patients who did not meet the UCSF criteria. Therefore, a total of 478 patients were included in our final analysis, and 1:3 PSM (with a caliper of 0.2) was conducted, yielding 256 eligible patients.

**Table 1** summarizes the basic demographic characteristics of the patients, laboratory parameters, and the histological and gross features of the tumors before and after PSM. Prior to PSM, the SLT group featured 77 patients (85.7% male, 14.3% female) with a median age of 48 years (range 18–67 years) and a median BMI of 22.0 kg/m² (range 15.0–34.0), The waiting time of the SLT group was 1-53 months, with an average waiting time of 30 months. The RH group featured 401 patients (86.8% male, 13.2% female) with a median age of 52 years (range 22–86 years) and a median BMI of 22.5 kg/m² (range 13.5–34.0). Significant differences were identified between the two groups in terms of hepatitis background, liver function parameters, platelet count, international normalized ratio, Child-Pugh classification, tumor number, and maximum tumor diameter (p <0.05). The SMD of the variables in the PSM is reduced to below 0.1, indicating that there is a good balance between the two groups. Following PSM, no significant differences were detected between the two groups, thereby enhancing data comparability. Compared with the RH group, the SLT group had more intraoperative blood loss, longer postoperative hospital stay, longer operation time, and more postoperative complications.

**Table 1:** Baseline characteristics and clinicopathological features of patients before and after propensity score matching.

Variables	Before propensity matching				After propensity matching			
	SLT (n=77)	RH (n=401)	*P-*Value	SMD	SLT (n=64)	RH (n=192)	*P-*Value	SMD
Age, years, median (range)	48 (18-67)	52 (22-86)	<0.001	0.378	49 (22-67)	52 (22-68)	0.106	0.081
Sexuality, n (%)								
Male	66 (85.7)	348 (86.8)	0.801	0.029	55 (85.9)	164 (85.4)	0.709	0.039
Female	11 (14.3)	53 (13.2)	0.855	0.018	9 (14.1)	28 (14.6)	1.000	0
BMI, kg/m^2^, median (range)	22.0 (15.0-34.0)	22.5 (13.5-34.0)	0.005	0.269	22.0 (15.0-33.0)	22.5 (13.8-33.8)	0.138	0.074
HBV, n (%)	66 (85.7)	332 (82.8)	0.827	0.022	56 (87.5)	167 (87.0)	0.565	0.048
HCV, n (%)	1 (1.3)	10 (2.5)	0.700	0.036	1 (1.6)	4 (2.1)	1.000	0
Alcohol, n (%)	2 (2.6)	2 (0.5)	0.124	0.091	1 (1.6)	2 (1.0)	1.000	0
Cirrhosis, n (%)	55 (71.4)	173 (43.1)	<0.001	0.524	42 (65.6)	108 (56.3)	0.241	0.062
ALT, IU/L, median (range)	34 (10-182)	35 (8-151)	0.408	0.059	35 (10-158)	34.5 (8-147)	0.560	0.049
AST, IU/L, median (range)	35.5 (17-200)	31 (15-180)	0.015	0.257	35 (19-192)	31 (25-168)	0.052	0.088
Platelet count, ×10^9^/L, median (range)	92 (30-286)	111 (22-302)	0.041	0.201	95 (31-239)	103 (27-254)	0.124	0.076
Total bilirubin, umol/L, median (range)	14.6 (3.0-50.1)	14.1 (3.8-31.8)	0.183	0.083	14.7 (3.0-45.5)	14.3 (3.8-31.0)	0.114	0.079
INR, median (range)	1.09 (0.87-2.45)	1.04 (0.84-1.58)	<0.001	0.361	1.08 (0.87-1.39)	1.05 (0.86-1.32)	0.103	0.082
Creatinine, umol/L, median (range)	70.9 (23.0-231.0)	72.3 (32.0-125.0)	0.274	0.072	71 (25-216)	73 (42-115)	0.720	0.036
ALB, g/L, median (range)	42.5 (28.3-54.0)	43.1 (31.5-56.9)	0.056	0.187	42.5 (28.6-53.2)	43.0 (31.7-52.8)	0.201	0.064
Child - Pugh score, median (range)	5 (5-12)	5 (5-12)	<0.001	0.446	5 (5-12)	5 (5-10)	0.265	0.058
Child - Pugh A/B/C, n (%)								
A	60 (77.9)	386 (96.3)	<0.001	0.482	53 (82.8)	177 (92.2)	0.198	0.065
B	12 (15.6)	15 (3.7)	<0.001	0.315	11 (17.2)	15 (7.8)	0.172	0.071
C	5 (6.5)	–						
MELD score, median (range)	7 (1-27)	7 (2-15)	0.452	0.052	7 (2-15)	7 (2-10)	0.514	0.056
Serum AFP ≥ 100 ng/mL, n (%)	26 (33.8)	147 (6.7)	0.594	0.044	24 (37.5)	76 (39.6)	0.897	0.022
Intraoperative blood loss, mL, median (range)	800 (300-2800)	300 (100-1200)	<0.001	0.423	800 (400-2400)	400 (100-800)	<0.001	0.215
Multiple tumors, n (%)	16 (20.8)	23 (5.7)	<0.001	0.429	11 (17.2)	21 (10.9)	0.196	0.068
Maximum tumor diameter≥5cm, n(%)	50 (64.9)	210 (52.4)	0.046	0.199	38 (59.4)	94 (49.0)	0.193	0.069
BCLC, n (%)								
0	7 (9.1)	62 (15.5)	0.161	0.086	7 (10.9)	35 (18.2)	0.602	0.046
A	64 (83.1)	328 (81.8)	0.782	0.032	55 (85.9)	149 (77.6)	0.727	0.035
B	4 (5.2)	4 (1.0)	0.026	0.242	1 (1.6)	4 (2.1)	1.000	0
C	2 (2.6)	7 (1.7)	0.642	0.039	1 (1.6)	4 (2.1)	1.000	0
Differentiation of HCC, n (%)								
Well	3 (3.9)	7 (1.7)	0.208	0.081	2 (3.1)	5 (2.6)	1.000	0
Moderate	46 (59.7)	233 (58.1)	0.802	0.028	40 (62.5)	126 (65.6)	0.547	0.051
Poor	28 (36.4)	161 (40.1)	0.611	0.041	23 (34.4)	61 (31.8)	0.542	0.052
Microvascular invasion, n (%)	21 (27.3)	76 (19.0)	0.121	0.092	20 (31.3)	57 (29.7)	0.875	0.025
ICU stay, d, median (range)	1 (1-23)	1 (1-5)	<0.001	0.412	3 (1-6)	2 (1-4)	0.149	0.072
Postoperative hospital stay, d, median (range)	15 (9-36)	8 (6-18)	<0.001	0.355	14 (10-30)	7 (6-15)	<0.001	0.211
Operation time min, median (range)	412 (302-487)	253 (189-352)	<0.001	0.312	380 (320-443)	235 (200-314)	<0.001	0.268
Postoperative complications occurred, n (%)	41 (53.2)	151 (37.7)	0.001	0.275	28 (43.8)	69 (35.9)	0.018	0.157
Tumor recurrence time ≤ 24 months, n (%)	35 (45.5)	110 (27.4)	0.061	0.176	30 (46.9)	102 (53.1)	0.386	0.048

SLT, salvage liver transplantation; RH, repeated hepatectomy; SMD, standardized mean differences; BMI, body mass index; HBV, hepatitis B virus; HCV, hepatitis C virus; ALT, alanine aminotransferase; AST, aspartate aminotransferase; INR, international normalized ratio; ALB, albumin; MELD, model for end-stage liver disease; AFP, alpha-fetoprotein; BCLC, Barcelona Clinic Liver cancer; ICU, intensive care unit.

### 3.2 Baseline data at initial resection

There were no significant differences between the SLT and RH groups in terms of hepatitis background, serum AFP levels (≥100 ng/mL), Child-Pugh classification, Barcelona Clinic Liver cancer (BCLC) staging, differentiation grade, microvascular invasion, tumor number, or maximum tumor diameter (p >0.05; **Table 2**). This indicated comparable postoperative outcomes between the two groups.

**Table 2:** Baseline characteristics and clinicopathological features of patients at the time of initial resection.

Variables	SLT (n=77)	RH (n=401)	*P-*Value
Age, years, median (range)	45 (16-65)	50 (20-71)	0.135
Sexuality, n (%)			
Male	66 (85.7)	348 (86.8)	0.801
Female	11 (14.3)	53 (13.2)	0.855
BMI, kg/m^2^, median (range)	22.5 (15.0-34.0)	22.5 (13.8-33.8)	0.774
HBV, n (%)	64 (83.1)	333 (83.3)	0.659
HCV, n (%)	1 (1.3)	10 (2.5)	1.000
Alcohol, n (%)	1 (1.3)	6 (1.5)	1.000
Cirrhosis, n (%)	38 (49.4)	185 (46.1)	0.620
ALT, IU/L, median (range)	33 (4-300)	37 (11-277)	0.014
AST, IU/L, median (range)	32.0 (17-274)	36.0 (15-245)	0.041
Platelet count, ×10^9^/L, median (range)	112.5(31-470)	117 (28-458)	0.136
Total bilirubin, umol/L, median (range)	23.7 (3.0-51.8)	23.6 (3.80-35.8)	0.832
INR, median (range)	1.08(0.87-1.33)	1.04(0.84-1.58)	0.001
Creatinine, umol/L, median (range)	71 (40-113)	73 (38-110)	0.212
ALB, g/L, median (range)	42.3 (27.2-53.2)	42.9 (25.5-52.4)	0.266
Child - Pugh score, median (range)	5 (5-7)	5 (3-7)	0.012
Child - Pugh A/B/C, n (%)			
A	75 (97.4)	390 (97.3)	0.190
B	2 (2.6)	3 (0.7)	0.190
MELD score, median (range)	7 (2-12)	7 (1-15)	0.105
Serum AFP ≥ 100 ng/mL, n (%)	32 (41.6)	269 (67.1)	<0.001
Multiple tumors, n (%)	15 (19.5)	51 (12.8)	0.149
Maximum tumor diameter≥5cm, n(%)	55 (71.4)	273 (68.1)	0.643
BCLC, n (%)			
0	7 (9.1)	40 (10.0)	1.000
A	62 (80.5)	312 (77.8)	0.704
B	5 (6.5)	34 (8.5)	0.655
C	3 (3.9)	11 (2.7)	0.484
Differentiation of HCC, n (%)			
Well	3 (3.9)	11 (2.7)	0.480
Moderate	47 (61.0)	253 (63.1)	0.797
Poor	27 (35.1)	137 (34.2)	0.896
Microvascular invasion, n (%)	18 (23.4)	78 (19.5)	0.439
ICU stay, d, median (range)	4 (1-14)	2 (1-13)	0.038

HCC, hepatocellular carcinoma; SLT, salvage liver transplantation; RH, repeated hepatectomy; BMI, body mass index; HBV, hepatitis B virus; HCV, hepatitis C virus; ALT, alanine aminotransferase; AST, aspartate aminotransferase; INR, international normalized ratio; ALB, albumin; MELD, model for end-stage liver disease; AFP, alpha-fetoprotein; BCLC, Barcelona Clinic Liver cancer; ICU, intensive care unit.

### 3.3 Analysis of survival outcomes

**Figure 2:**
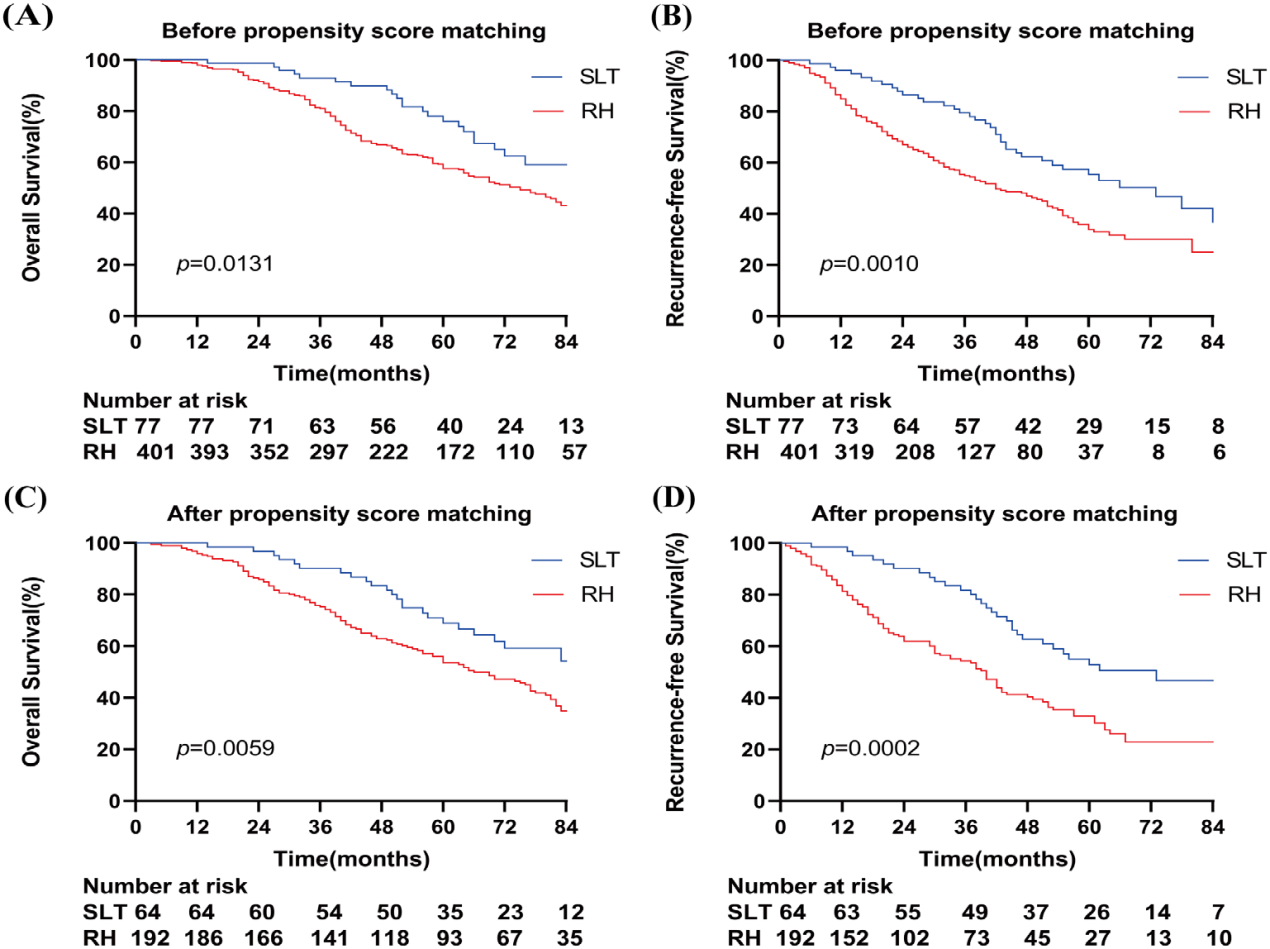
Overall survival **(A)** and relapse-free survival **(B)** of patients who received SLT or RH prior to propensity score matching. Overall survival **(C)** and recurrence-free survival **(D)** of patients who received SLT or RH after propensity score matching. SLT, salvage liver transplantation; RH, repeated hepatectomy.

The median survival time after recurrence in patients who underwent SLT and RH was 94 and 75 months, respectively. The 1-, 3-, and 5-year OS rates in the SLT group were 100%, 93.0%, and 76.1%, respectively, compared with 98.0%, 81.0%, and 57.6% in the RH group, respectively (p = 0.0131) (**Figure 2A**). The median recurrence time was 73 and 42 months in the SLT and RH groups, respectively. The 1-, 3-, and 5-year RFS rates were 96.0%, 79.4%, and 55.4% in the SLT group and 84.9%, 54.9%, and 33.9% in the RH group, respectively (p = 0.0010; **Figure 2B**), indicating significant differences between the two groups in terms of OS and RFS.

After PSM, the median survival times in the SLT and RH groups were 86 and 66 months, respectively. The 1-, 3-, and 5-year OS rates in the SLT group were 100%, 90.2%, and 68.9%, respectively, compared with 95.8%, 75.3%, and 53.6% in the RH group, respectively (p = 0.0059) (**Figure 2C**). The median recurrence time was 73 months for SLT and 40 months for RH, with 1-, 3-, and 5-year RFS rates of 98.4%, 81.7%, and 53.9% in the SLT group, and 81.4%, 54.4%, and 30.3% in the RH group, respectively (p = 0.0002) (**Figure 2D**). These results indicate significant differences between the two groups in terms of OS and RFS.

### 3.4 Risk factor analysis for survival outcomes

Univariate Cox regression analysis revealed that cirrhosis(HR: 1.785, 95% CI: 1.254-2.541, p=0.001), Child-Pugh grade B/C(HR: 2.496, 95% CI: 1.619-3.851, p<0.001), serum AFP levels ≥100 ng/mL(HR: 1.484, 95% CI: 1.049-2.099, p=0.026), multiple tumors(HR: 1.942, 95% CI: 1.249-3.018, p=0.003), maximum tumor diameter ≥5 cm(HR: 1.659, 95% CI: 1.188-2.318, p=0.003), BCLC stage B/C(HR: 1.962, 95% CI: 2.180-16.308, p=0.001), Poor differentiation of HCC(HR: 1.941, 95% CI: 1.380-2.730, p<0.001), microvascular invasion(HR: 3.828, 95% CI: 2.709-5.407, p<0.001), Tumor recurrence time ≤ 24 months(HR:3.571, 95% CI: 2.520-5.060, p<0.001), and Operative method (RH)(HR: 2.034, 95% CI: 1.512-2.738, p=0.006) were significantly linked to a poor prognosis (p <0.05; **Table 3**). Multivariate analysis identified multiple tumors (HR: 1.745, 95% CI: 1.054–2.891, p=0.031), maximum tumor diameter ≥5 cm (HR: 1.520, 95% CI: 1.050–2.200, p=0.027), microvascular invasion (HR: 2.697, 95% CI: 1.785–4.073, p <0.001), Tumor recurrence time ≤ 24 months (HR: 2.311, 95% CI: 1.532–3.485, p <0.001), and Operative method (RH) (HR: 1.611, 95% CI: 1.281–2.233, p=0.034) as independent risk factors. Using patients who did not undergo repeat liver resection as a reference, undergoing repeat liver resection was identified as an independent risk factor.

**Table 3:** Univariate and multivariate analysis of prognostic factors for survival.

Variables	Before propensity matching			After propensity matching		
	HR	95%CI	*P-*Value	HR	95%CI	*P-*Value
Univariate analysis						
Age	1.001	0.984-1.018	0.906	1.077	0.716-1.620	0.721
Sexuality	0.982	0.606-1.593	0.943	1.063	0.675-1.674	0.793
HBV	1.247	0.689-2.259	0.466	1.506	0.849-2.674	0.162
HCV	1.258	0.399-3.969	0.695	1.029	0.327-3.327	0.961
Cirrhosis	1.939	1.353-2.777	<0.001	1.785	1.254-2.541	0.001
Child - Pugh B/C	2.250	1.472-3.439	<0.001	2.496	1.619-3.851	<0.001
MELD score≥9	1.471	0.858-2.522	0.161	1.603	0.904-2.841	0.106
Serum AFP ≥ 100 ng/mL	1.439	1.015-2.039	0.041	1.484	1.049-2.099	0.026
Multiple tumors	1.771	1.131-2.772	0.012	1.942	1.249-3.018	0.003
Maximum tumor diameter≥5cm	1.449	1.038-2.024	0.029	1.659	1.188-2.318	0.003
BCLC stage B/C	2.307	1.936-14.547	0.001	1.962	2.180-16.308	0.001
Poor differentiation of HCC	2.144	1.523-3.019	<0.001	1.941	1.380-2.730	<0.001
Microvascular invasion	3.196	2.269-4.501	<0.001	3.828	2.709-5.407	<0.001
Tumor recurrence time ≤ 24 months	4.918	3.438-7.035	<0.001	3.571	2.520-5.060	<0.001
Operative method (RH)	1.539	1.077-2.153	0.017	2.034	1.512-2.738	0.006
Multivariate analysis						
Cirrhosis	1.356	0.906-2.029	0.138	1.223	0.821-1.821	0.323
Child - Pugh (B/C)	1.167	1.338-3.508	0.202	1.202	1.010-1.892	0.124
Serum AFP ≥ 100 ng/mL	1.264	0.872-1.832	0.215	1.333	0.921-1.930	0.128
Multiple tumors	1.815	1.105-2.981	0.019	1.745	1.054-2.891	0.031
Maximum tumor diameter≥5cm	1.411	1.089-2.063	0.042	1.520	1.050-2.200	0.027
BCLC (B/C)	1.354	0.460-3.986	0.583	1.453	0.486-4.344	0.504
Differentiation of HCC (poor)	1.427	0.981-2.077	0.113	1.210	0.826-1.775	0.328
Microvascular invasion	1.754	1.167-2.635	0.007	2.697	1.785-4.073	<0.001
Tumor recurrence time ≤ 24 months	3.396	2.218-5.200	<0.001	2.311	1.532-3.485	<0.001
Operative method (RH)	1.611	1.281-2.233	0.025	1.625	1.211-2.414	0.034

HR, hazard risk; CI, confidence interval; HBV, hepatitis B virus; HCV, hepatitis C virus; MELD, model for end-stage liver disease; AFP, alpha-fetoprotein; BCLC, Barcelona Clinic Liver cancer.

### 3.5 Subgroup analysis based on risk factors

**Figure 3:**
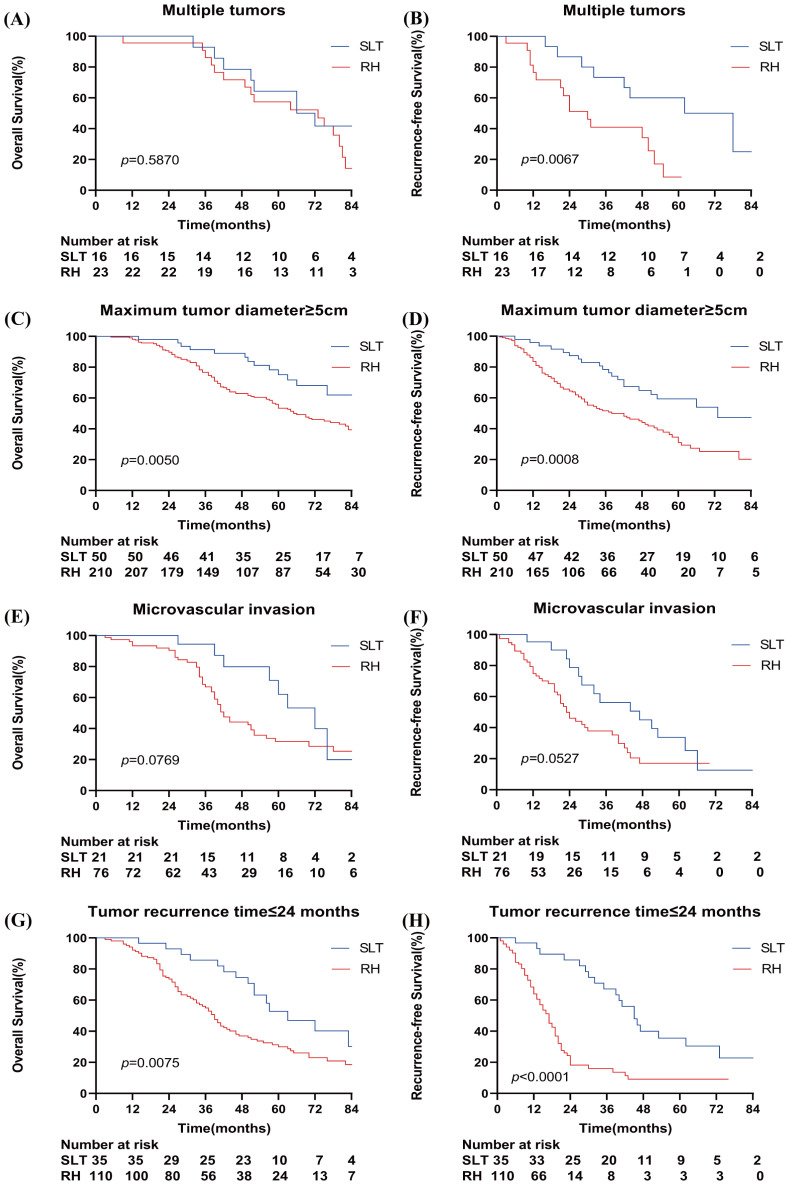
Kaplan–Meier curves for overall survival and recurrence-free survival in the study cohort when stratified by multiple tumors **(A, B)**, a total tumor length ≥5 cm **(C, D)**, microvascular invasion **(E, F)**, and a tumor recurrence time ≤24 months **(G, H)**. SLT, salvage liver transplantation; RH, repeated hepatectomy.

Subgroup analysis revealed that there were no significant differences in the 1-, 3-, and 5-year OS rates between the SLT and RH groups in patients with multiple tumors (100%, 92.9%, and 64.3% vs. 95.7%, 86.1%, and 57.4%, respectively; p=0.5870) (**Figure 3A**). In patients with a maximum tumor diameter ≥5 cm, the SLT group demonstrated significantly higher OS rates than the RH group (100%, 91.4%, and 75.1% vs. 98.1%, 76.7%, and 53.4%, respectively; p=0.0050) (**Figure 3C**). No significant differences were observed in terms of OS rates between the SLT and RH groups for patients with microvascular invasion (100%, 94.4%, and 62.2% vs. 93.4%, 66.7%, and 31.7%, respectively; p=0.0769) (**Figure 3E**). In patients with a recurrence time ≤2 years, the SLT group had significantly higher OS rates than the RH group (100%, 85.7%, and 52.8% vs 92.2%, 55.0%, and 30.0%; p=0.0075) (**Figure 3G**).

When considering patients with multiple tumors, the SLT group exhibited significantly higher RFS rates than the RH group (100%, 73.3%, and 60.0% vs. 76.5%, 41.0%, and 8.6%, respectively; p=0.0067) (**Figure 3B**). The SLT group had significantly higher RFS rates than the RH group in patients with a maximum tumor diameter ≥5 cm (95.9%, 78.5%, and 59.4% vs 83.6%, 51.6%, and 31.1%; p=0.0008) (**Figure 3D**). No significant differences in RFS rates were detected between the SLT and RH groups in patients with microvascular invasion (95.2%, 56.2%, and 33.7% vs. 74.8%, 37.9%, and 17.1%, respectively; p=0.0527) (**Figure 3F**). In patients with a recurrence time ≤2 years, the SLT group demonstrated significantly higher RFS rates than the RH group (96.7%, 67.1%, and 35.5% vs 64.0%, 15.9%, and 9.1%; p<0.0001) (**Figure 3H**).

### 3.6 Outcomes analysis based on a risk score model

**Figure 4:**
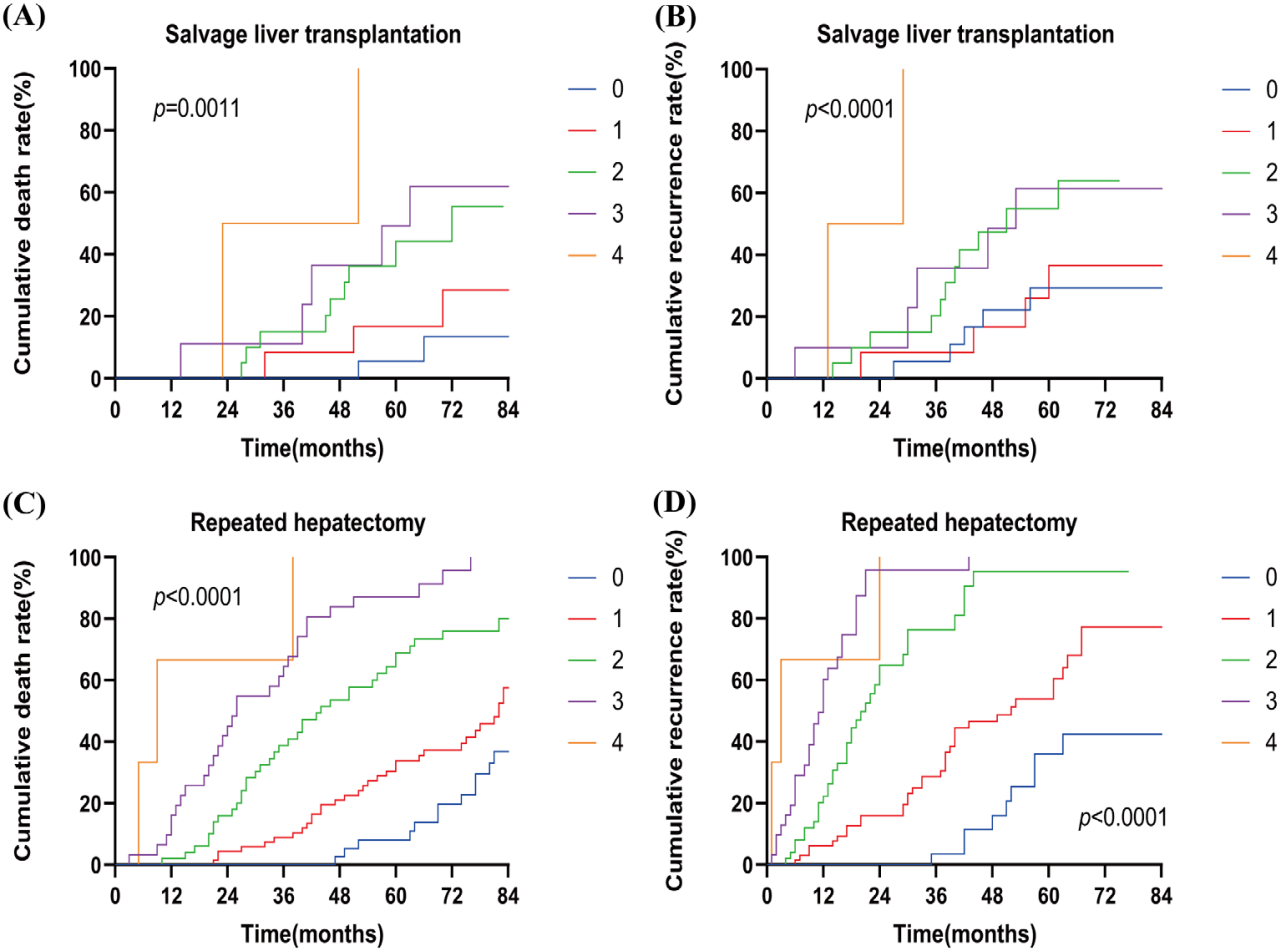
Cumulative death rate **(A)** and cumulative recurrence rate **(B)** of patients with different numbers of risk factors in SLT. Cumulative death rate **(C)** and cumulative recurrence rate **(D)** for patients with different numbers of risk factors in RH. SLT, salvage liver transplantation; RH, repeated hepatectomy; 0–4, the number of risk factors.

As shown in **Figure 4**, in the SLT group, the cumulative mortality and recurrence rates were significantly higher when two risk factors (n=2) were present compared to one risk factor (n=1). However, there was no significant increase in cumulative mortality and recurrence rates between 0 and 1 risk factors (n=0, n=1). Similarly, no significant increase in cumulative mortality and recurrence rates was observed between 2 and 4 risk factors (n=2, n=3, n=4).In the RH group, cumulative mortality and recurrence rates increased progressively with the number of risk factors.

**Figure 5:**
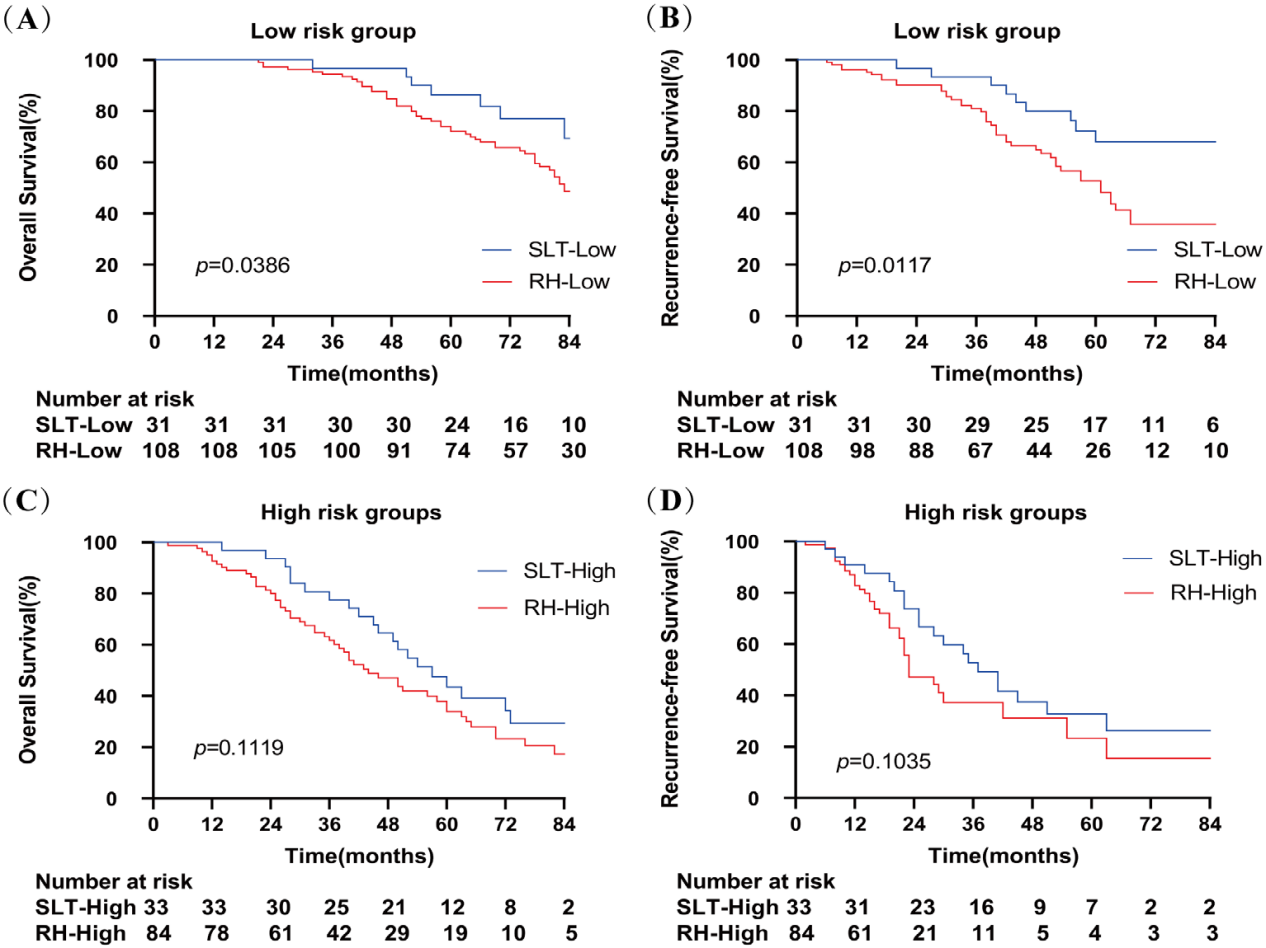
Overall survival **(A)** and recurrence-free survival **(B)** of patients receiving SLT or RH in the low-risk group. Overall survival **(C)** and recurrence-free survival **(D)** of patients receiving SLT or RH in the high-risk group. SLT, salvage liver transplantation; RH, repeated hepatectomy.

Next, we defined zero and one risk factor as a low-risk group and two to four risk factors as a high-risk group. **Figure 5** shows that SLT patients had significantly higher OS rates than RH patients in the low-risk group (100%, 96.7%, and 86.4% vs. 100%, 94.4%, and 72.0%, respectively; p=0.0386). In the high-risk group, OS rates were comparable between the SLT and RH groups (100%, 77.5%, and 43.5% vs. 92.7%, 61.7%, and 33.9%, respectively; p=0.1119). In the low-risk group, patients with SLT exhibited significantly higher RFS rates than RH patients (100%, 83.6%, and 40.3% vs. 89.5%, 53.7%, and 16.1%, respectively; p=0.0117). In the high-risk group, RFS rates were comparable between the SLT and RH groups (90.9%, 52.7%, and 32.8% vs. 82.8%, 37.3%, and 23.3%, respectively; p =0.1035).

## 4 Discussion

SLT and RH are currently considered as effective treatments for patients with rHCC (21–23). While SLT provides longer survival and lower recurrence rates when compared to RH, its clinical application is constrained by donor shortages, the risk of tumor progression resulting in dropout from waiting lists, and strict transplant criteria (7, 15). To optimize donor resource utilization and prevent unnecessary wastage, the choice between SLT and RH for rHCC should consider patient demographic characteristics, laboratory parameters, and tumor pathological staging prior to recurrence.

When considering patients with rHCC who met the UCSF criteria, we found that the SLT group had a 5-year OS of 76.1%, a 5-year RFS of 55.4%, a median survival of 94 months, and a median recurrence interval of 73 months. In comparison, the RH group had a 5-year OS of 57.6%, a 5-year RFS of 33.9%, a median survival of 75 months, and a median recurrence interval of 42 months (**Figure 2A**). Thus, the SLT group exhibited longer survival and lower recurrence rates; these findings were consistent with those reported by Fang et al. (5-year OS, 77.1%; RFS, 81.2% in the SLT group; 5-year OS, 55.6%; and RFS, 36.9% in the RH group) (24). Similarly, previous studies confirmed that SLT outperformed RH (15, 16, 25, 26). These previous studies primarily involved patients with rHCC who met the Milan criteria and initially underwent LR or RFA. Our present study extended these findings to the UCSF criteria, thus broadening transplantation opportunities for a wider range of patients.

Cox proportional hazards regression analysis further identified tumor number, maximum tumor diameter, microvascular invasion, and recurrence time, as independent risk factors for survival and recurrence. Multiple nodules and large tumors are frequently associated with highly invasive biological behaviors and an elevated risk of tumor progression in HCC (11). RFA is known to achieve favorable survival outcomes when the number of tumors is ≤3, the maximum tumor diameter is < 5 cm, and there is no microvascular invasion (20). Research has shown that in liver cancer patients, when the tumor diameter exceeds 5 cm, the degree of tumor invasion, survival time, and risk of recurrence significantly increase, and the likelihood of metastasis is also higher (27). In a previous study, Tsilimigras et al. demonstrated that the Tumor Burden Score (TBS, defined as the combination of tumor number and maximum tumor diameter) was able to predict the prognosis of patients with HCC undergoing LR, both within and beyond the Milan criteria (28). In another study, Moris et al. confirmed that the TBS could predict the prognosis of patients with HCC undergoing LT beyond the Milan criteria (29). Our present study utilized the UCSF criteria and yielded similar results, thus demonstrating the significant impact of tumor number and maximum tumor diameter on prognosis.

Microvascular invasion is known to exert a significant impact on both survival and recurrence in patients with HCC (30, 31). Previously, Lei et al. demonstrated that tumor number and maximum diameter represent key predictors for the risk of microvascular invasion (32). Similarly, in the present study, we found that patients with rHCC with microvascular invasion had a poorer prognosis, and that both tumor number and maximum diameter represented significant prognostic factors.

Choi et al. demonstrated that the timing of HCC recurrence after surgery significantly influenced survival, with early recurrence linked to poorer outcomes (33). Studies by Hu et al. and Lee et al. further support this conclusion (34, 35). In the present study, we identified a median recurrence time of 26 months (for the SLT group) and 23 months (for the RH group), with two years as a cut-off value, thus indicating a poor prognosis for patients who experience recurrence within two years. This finding aligns with previous studies that defined early recurrence as occurring within 2 years (36). Therefore, treatment strategies for the early recurrence of HCC should be carefully evaluated.

Based on Cox proportional hazards regression analysis, we stratified patients into low-risk (zero to one risk factor) and high-risk (two to four risk factors) groups. Low-risk patients who underwent SLT exhibited significantly higher OS and RFS rates than those who underwent RH (**Figure 5**). In contrast, when considering the high-risk patients, there was no significant difference in OS or RFS between those who underwent SLT and those who underwent RH. Thus, we recommend RH as the preferred option when two or more risk factors are present, and SLT for patients with zero to one risk factors to optimize donor use. At the same time, the difficulty of the SLT procedure and its impact on postoperative prognosis should also be taken into account.

The MELD score is widely used to evaluate the severity of liver disease and guide the allocation of organs. Furthermore, the MELD score possesses significant clinical value for predicting patient outcomes following organ transplantation. However, the MELD score has certain limitations (37). In that it benefits patients with more severe conditions but can increase early post-transplant mortality, potentially leading to organ wastage. Therefore, developing an organ allocation system based on more comprehensive evidence could enable stricter outcome evaluation and reduce unnecessary organ waste (38). In addition, several studies reported that factors such as time to recurrence after LR, as well as tumor size and number, could exert significant effects on patient prognosis (39–41). These findings are consistent with those of the present study. When considering patients with rHCC who met the UCSF criteria and underwent SLT after recurrence, we observed that those with multiple high-risk factors had similar outcomes regardless of whether they received SLT or RH. Therefore, we recommend that clinicians should consider these specific risk factors for organ allocation in patients with rHCC awaiting transplantation.

This study had certain limitations. First, as a retrospective study based in a single-center, there was potential for selection bias. Furthermore, the study population, consisting entirely of Chinese patients, may have introduced geographical variability. Second, as a non-randomized controlled study, and despite the use of PSM to reduce inter-group bias, some differences may have remained in the data when comparing between surgical options. Third, this study may have overlooked certain confounding factors related to prognosis. Finally, we did not statistically analyze postoperative treatment plans, potentially affecting our final results.

## 5 Conclusion

In conclusion, when performing SLT for rHCC, factors such as the number of tumors, maximum tumor diameter, microvascular invasion, and time to recurrence should be considered as risk factors. For patients with 0-1 risk factors, SLT is likely to yield better therapeutic outcomes compared to RH. However, for patients with 2-4 risk factors, RH may provide similar treatment outcomes to SLT. This conclusion could be beneficial for optimizing donor organ allocation.

## Data Availability

The original contributions presented in the study are included in the article/supplementary material. Further inquiries can be directed to the corresponding authors.
